# Periocular Manifestation of Obstructive Sleep Apnea as a Novel Perioperative Screening Tool

**DOI:** 10.1007/s11695-021-05851-7

**Published:** 2022-01-29

**Authors:** Megan Folsom, James Gigantelli, Brent Timperley, Kurtis Johnson, Danstan Bagenda, Huiling Pang, Sheila Ellis

**Affiliations:** 1 Department of Anesthesiology, University of Nebraska Medical Center, 42nd and Emile, Omaha, NE 68198 USA; 2 Present Address: Department of Anesthesiology, University of Kansas Medical Center, 3901 Rainbow Blvd, MS 1034, Kansas City, KS 66160 USA; 3 Department of Ophthalmology, Marshall University, 1 John Marshall Drive, Huntington, WV 25755 USA; 4 Department of Ophthalmology, University of Nebraska Medical Center, 42nd and Emile, Omaha, NE 68198 USA

**Keywords:** Floppy eyelid syndrome, Eyelid laxity, Obstructive sleep apnea, Screening tool, Perioperative medicine, Preoperative assessment

## Abstract

**Purpose:**

Obstructive sleep apnea (OSA) presents perioperative challenges with increased risk for complications. Floppy eyelid syndrome (FES) is associated with OSA yet has not been addressed perioperatively. The current standard for perioperative OSA screening includes assessing patient risk factors or the STOP-BANG tool, which requires an active participant. We aimed to confirm a connection between FES and OSA in presurgical patients and develop a screening method appropriate for patients with perioperative OSA risk.

**Materials and Methods:**

162 presurgical pre-anesthesia clinic patients were enrolled. Screening questions determined eligibility. Those who were pregnant or aged < 19 were excluded. Control group included those with a STOP-BANG score < 3. Experimental group included those with BMI > 35 and OSA diagnosis. Examiners photographed participants’ eyes with vertical and horizontal retraction while two blinded ophthalmologists used a grading scale to review grade of eyelid laxity.

**Results:**

Differences in habitus, ASA score, and hypertension as a comorbidity were significant. Sensitivity of FES screening was 52% (CI 37–66%) and specificity was 56% (CI 46–66%) for reviewer 1. For reviewer 2, sensitivity was 48% (CI 28–69%) and specificity was 72% (CI 60–81%). Negative predictive value was 86% (CI 81–90) for reviewer 1 and 88% (CI 83–92%) for reviewer 2. Inter-rater agreement was moderate.

**Conclusion:**

While specificity and sensitivity were lower than anticipated, negative predictive value was high. Given this strong negative predictive value, our findings indicate using eyelid retraction to screen for FES has perioperative clinical utility. These findings encourage further research addressing the connection of lid laxity/FES to OSA.

**Key Points:**

• Aimed to investigate if a FES screening tool could identify perioperative OSA risk.

• Negative predictive value for FES with OSA was 86%.

• Observing periocular lid laxity has clinical utility; is feasible in any patient.

**Graphical abstract:**

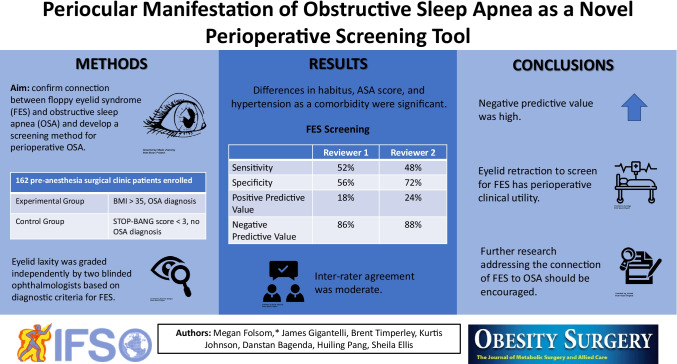

## Introduction

Obstructive sleep apnea (OSA) challenges anesthesiologists in perioperative settings. Those with OSA can experience increased sensitivity to narcotics, airway complications, cardiac arrest, and anoxic brain injury, with increased risk for serious perioperative complications [1, 2]. A meta-analysis found the prevalence of OSA in the general population ranged from 9 to 38%, increasing with age and obesity [3]. OSA may also occur with hypertension, diabetes, and metabolic syndrome [1]. OSA with or without these comorbidities can present as periocular manifestations [1].

In epidemiological studies, the mean prevalence of OSA was 22% in men and 17% in women when defined as an apnea–hypopnea index (AHI) ≥ 5 [4]. In addition, Chan et al. found that nearly 68% of patients undergoing major noncardiac surgery had unrecognized OSA with increased risk of 30-day postoperative cardiovascular complications based on preoperative oximetry sleep studies [5]. While exact prevalence is unknown and varies depending on the study, OSA can present at lower rates in community-screened populations and higher rates in certain subgroups [6]. The literature regarding the effect of OSA on perioperative outcomes implies that patients with undiagnosed or untreated OSA may experience an acute perioperative event after receiving an anesthetic that may exacerbate OSA. Medications used in the administration of anesthesia relax upper airway structures, leading to obstruction. They also affect lung mechanics, ventilation, oxygenation, and airway protection, all of which can exacerbate OSA or cause acute airway obstruction perioperatively. Thus, preoperatively identifying patients with or at risk for OSA can be of importance.

Floppy eyelid syndrome (FES), a periocular manifestation, was originally described in 1981 [7]. Excessive glycosaminoglycan deposition in the periorbital region results from prolonged ischemia, inflammation, and reperfusion injury consistent with OSA. These depositions lead to profound periocular soft tissue laxity, which predispose the eyelids to easy distraction and outward rotation from the ocular surface, especially during sleep.

Studies [8–12] have shown a positive association between FES and OSA, but have not addressed association within the perioperative domain [10] or screening tool development for FES as a predictor for OSA as a predictor for perioperative complications. Standard perioperative screening includes assessing risk factors such as snoring, tiredness, observed apnea, high blood pressure, body mass index (BMI), age, neck circumference, and male gender. The STOP-BANG tool [13] is also used, which despite high sensitivity requires appropriate communication skills, an awake patient or guardian, and a known health history. It also requires provider time to measure the patient’s neck and ask eight screening questions. We aimed to confirm a connection between FES and OSA in presurgical patients and develop a fast and reliable screening method that can be used on any patient to identify perioperative OSA.

## Methods

### Study Participants

Patients referred to the pre-anesthesia surgical clinic (PASC) prior to surgical intervention between 29 August 2017 and 25 January 2018 were considered for study participation. All patients were at least 19 years, which is the age of consent where the study was conducted. Overall OSA prevalence in the PASC was based on problem list data over a 6-month period of 457/2855 patients (16%). Following Institutional Review Board approval, all study participants provided written informed consent prior to intervention. Patients completed screening questions determining medical history and risk factors for OSA. A STOP-BANG score was calculated for patients with no history of OSA. The participants were then divided into two groups. The OSA group included non-pregnant adults over 19 years old who were evaluated preoperatively in the PASC with a formal diagnosis of OSA and a BMI greater than 35. The non-OSA group included nonpregnant adults over 19 years old who were evaluated preoperatively in the PASC with no formal diagnosis of OSA and a STOP-BANG score less than 3. The validated STOP-BANG questionnaire has high sensitivity in surgical populations with pooled sensitivities to predict any OSA (84%), moderate-to-severe OSA (91%), and severe OSA (96%) [14]. Thus, patients with STOP-BANG scores less than 3 were considered low risk of OSA and used as controls. Participants diagnosed with FES during the study were contacted, informed of the potential association with OSA, and encouraged to discuss with their primary care physician.

### Ophthalmologic Examination

**Fig. 1:**
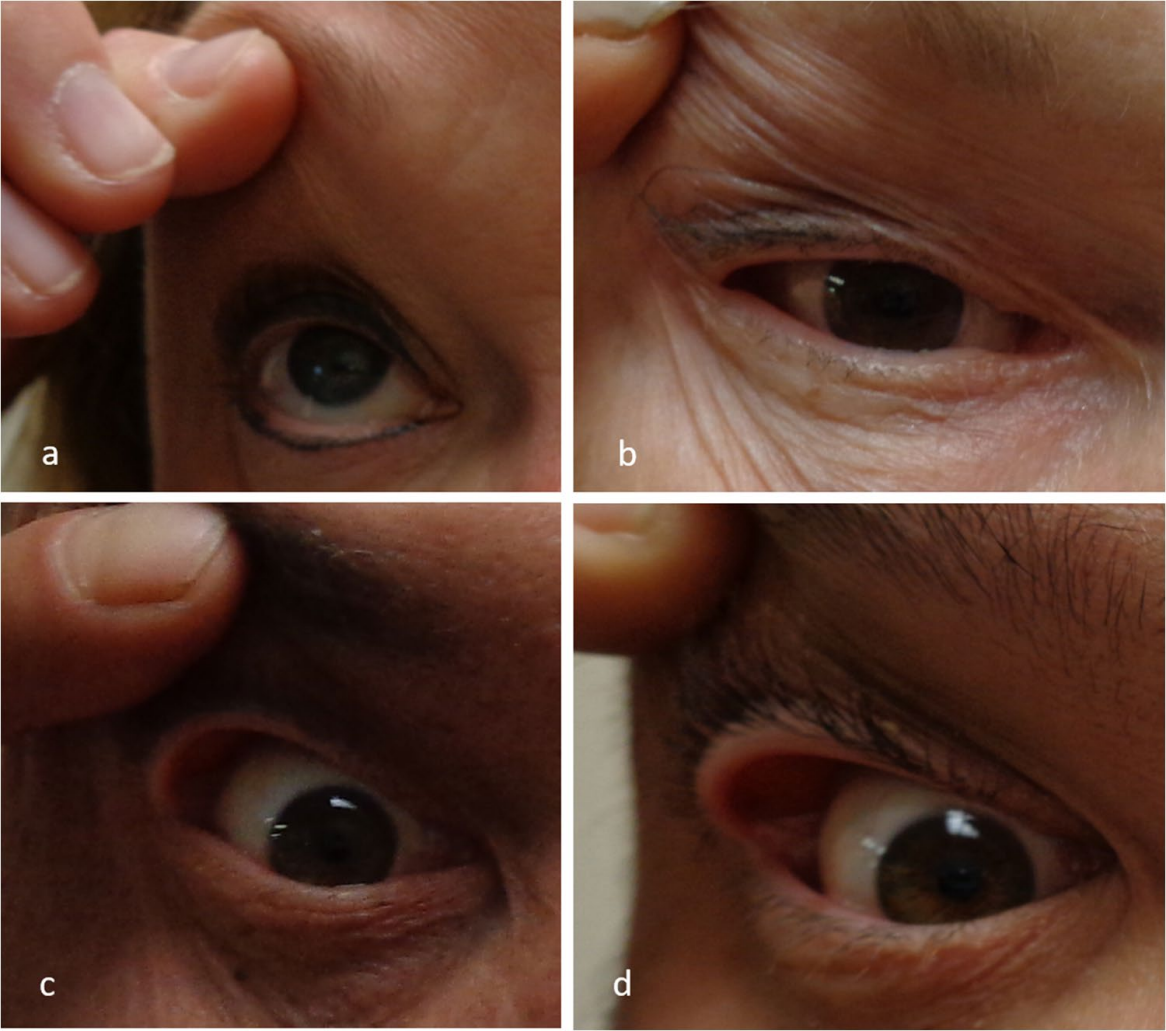
Examples of the types of lid laxity graded within the study: **a** normal laxity; **b** mild laxity; **c** moderate laxity; **d** severe laxity

Since ophthalmologists were not readily available in the PASC to assess lid laxity and diagnose FES, the following procedure was utilized to assess eyelid laxity: Participants held a cardboard cover up to the bridge of their nose (to obscure the face below the eyes), the examiner applied mild horizontal tension at the lateral canthus, and a photograph was taken on a digital camera while tension was applied. The examiner then applied mild vertical tension on the superior eyelid and another photograph was taken. The same procedure was performed on the contralateral eye. The pictures were taken from a standardized length of 2 feet from the subject’s face. The photographs were evaluated independently by two ophthalmologists who were blinded to the patient histories as well as each other’s interpretations of the photographs. Eyelid laxity was graded for each photograph based on the diagnostic criteria for FES described by Chambe et al. [8] in Table 1. Grade 1 represents the clinical definition of lax eyelid syndrome. Grade 2 and higher represent the clinical definition of FES. Grade 4 was not assessed by photograph in the present study. Any photograph with grade 2 or 3 was considered diagnostic of FES in the patient. Figure 1 shows examples of the types of lid laxity graded within this study.

**Table 1:** Diagnostic criteria for floppy eyelid syndrome

Grade 0	Grade 1	Grade 2	Grade 3	Grade 4
Normal laxity	Asymptomatic upper eyelid hyperlaxity (clinical definition of lax eyelid syndrome)	Papillary conjunctivitis with eyelid hyperlaxity (clinical definition of floppy eyelid syndrome)	Grade 2 + tarsal eversion when the eyelid is horizontally retracted	Grade 3 + persisting tarsal eversion with release of the eyelid

Chambe et al. [8]

### Statistical Analysis

Descriptive statistics were computed for all variables to ensure data quality and to evaluate the assumptions of statistical tests. Subgroup comparisons between those with OSA and those without were also performed on key demographic and medical history variables with the *χ*^2^ test, *T* test for means, and Wilcoxon test for medians. *P* values less than 0.05 were considered significant. When presenting results, an asterisk was used to note where Fisher’s exact test was utilized. The χ^2^ test was used to compare the proportion of patients with FES (grade 2 or 3 in any photograph) between the OSA and non-OSA groups. Sensitivity and specificity, and their respective 95% CIs, were determined for the FES-screening tool with a diagnostic cut-off of grade 2 in any patient image [14]. Positive predictive values (PPVs), negative predictive values (NPVs), and likelihood ratios were also calculated. These parameters were determined separately for each ophthalmologist’s evaluations to analyze inter-rater reliability. Agreement between the two evaluators in diagnosing FES based on the image sets was assessed by the kappa statistic. De-identified patient data from the PASC clinic was utilized to determine prevalence of OSA in the population being studied. With an estimated 30% prevalence of OSA in the study population, the minimum sample size to achieve 80% power and detect a difference between group proportions of 0.26 was determined to be 163, with at least 49 having an OSA diagnosis.

## Results

In total, 162 patients were included: 50 with a formal diagnosis of OSA by sleep study (OSA group) and 112 with no history of OSA and STOP-BANG less than 3 (non-OSA group). Of the 162 participants, 157 image sets were evaluated by ophthalmologists and included in the statistical analysis. Four patient images were lost due to camera memory card malfunction and one patient image was of inadequate clarity for evaluation. One patient in the non-OSA group was excluded from data analysis due to self-reported history of OSA and continuous positive airway pressure (CPAP) use.

Participant demographics, comorbidities, risk factors, and screening responses are shown in Table 2. As expected, weight was significantly higher in the OSA group with a mean weight of 123.62 kg (SD 24.98) versus a non-OSA group mean weight of 78.9 kg (SD 17.74) (*p* < 0.001). Average American Society of Anesthesiology (ASA) score was significantly higher in the OSA group (*p* = 0.03). Of the comorbidities analyzed, only hypertension was significantly more prevalent in the OSA group than the non-OSA group (*p* = 0.001).

**Table 2:** Demographics

	OSA negative—control group (*n* = 112)	OSA positive—experimental group (*n* = 50)	*p* value
Age mean (SD)	57.06 (16.24)	56.92 (11.63)	0.955
Age median (IQR)	59.00 (46.50, 69.25)	58.50 (48.00, 66.00)	0.707
Height mean cm (SD)	167.47 (9.03)	170.97 (10.55)	0.032
Height median cm (IQR)	165.75 (161.30, 172.70)	169.60 (162.60, 177.80)	0.057
Weight mean kg (SD)	78.69 (17.74)	123.62 (24.98)	< 0.001
Weight median kg (IQR)	76.45 (66.10, 89.70)	120.90 (103.40, 134.42)	< 0.001
BMI mean (SD)	27.55 (6.03)	41.70 (7.29)	< 0.001
BMI median (IQR)	26.00 (23.00, 31.00)	40.00 (36.00, 44.75)	< 0.001
Female *n* (%)	86 (76.8)	29 (58.0)	0.025
Race *n* (%)			0.186*
Caucasian, white	101 (90.2)	44 (88.0)	
African American	10 (8.9)	3 (6.0)	
Hispanic/Latino	1 (0.9)	2 (4.0)	
Asian, Pacific Islander	0 (0.0)	0 (0.0)	
Native American	0 (0.0)	1 (2.0)	
Other	0 (0.0)	0 (0.0)	
STOP-BANG score *n* (%)			< 0.001*
0	13 (11.6)	0 (0.0)	
1	39 (34.8)	0 (0.0)	
2	60 (53.6)	0 (0.0)	
3	0 (0.0)	0 (0.0)	
4	0 (0.0)	0 (0.0)	
5	0 (0.0)	0 (0.0)	
6	0 (0.0)	0 (0.0)	
7	0 (0.0)	1 (33.3)	
8	0 (0.0)	2 (66.7)	
ASA score *n* (%)			0.033*
1	1 (0.9)	0 (0.0)	
2	21 (18.8)	2 (4.0)	
3	78 (69.6)	44 (88.0)	
4	12 (10.7)	4 (8.0)	
5	0 (0.0)	0 (0.0)	
Comorbidities *n* (%):			
CAD	9 (8.0)	9 (18.0)	0.111
CHF	3 (2.7)	5 (10.0)	0.111
DM	18 (16.1)	13 (26.0)	0.205
COPD	14 (12.5)	5 (10.0)	0.847
TIA/CVA	9 (8.0)	2 (4.0)	0.545
Hyperlipidemia	29 (25.9)	19 (38.0)	0.170
Hypertension	53 (47.3)	39 (78.0)	0.001
Renal insufficiency	11 (9.8)	5 (10.0)	1.000
Cancer	23 (20.5)	8 (16.0)	0.644
Patient history *n* (%)			
Smoking history	60 (53.6)	25 (50.0)	0.802
Drug abuse	4 (3.6)	4 (8.0)	0.426
Alcohol use	49 (44.1)	21 (43.8)	1.000
Obstructive sleep apnea	1 (0.9)	50 (100.0)	< 0.001
Home CPAP use	1 (0.9)	46 (92.0)	< 0.001
Current home narcotic use	18 (16.4)	6 (12.0)	0.633

^*^Fisher’s exact test

The true sample prevalence of OSA in the patients who underwent ophthalmic examination for reviewer 1 was 32% (CI 25–40) and for reviewer 2 was 25% (CI 17–35). FES was diagnosed in 52% of the patients in the OSA group and 44% of the patients in the non-OSA group using a diagnostic cut-off of grade 2 or higher lid laxity in either horizontal or vertical traction in either eye.

Regarding FES picture screening characteristics, the results between the two investigating ophthalmologists were very similar with a computed agreement of 76%. After adjustment for chance, Cohen’s kappa was determined to be 53%, indicating moderate inter-rater agreement (41–60% considered moderate). Using the FES diagnostic cut-off of grade 2 or higher lid laxity, sensitivity for the FES-based examination was 52% (CI 37–66) and 48% (CI 28–69) for reviewer 1 and 2, respectively. Specificity was 56% (CI 46–66) and 72% (CI 60–81) for reviewer 1 and 2, respectively. Likelihood Ratio + was calculated at 1.18 (95% CI = 0.84, 1.67) and Likelihood Ratio − was calculated to be 0.86 (95% CI = 0.61, 1.20). Lid laxity for FES had a positive predictive value of 18% (CI 14–24) for reviewer 1 and 24% (CI 16–36) for reviewer 2. A negative predictive value of 86% (CI 81–90) was seen for reviewer 1 and 88% (CI 83–92) for reviewer 2. Reviewer outcomes are summarized in Table 3 and an inter-rater reliability table is referenced in Table 4.

**Table 3:** Reviewer outcomes

	Reviewer 1 Point estimates (95% CIs)	Reviewer 2 Point estimates (95% CIs)
Estimated facility prevalence	0.16	0.16
Detection rate	0.17	0.12 (0.07, 0.21)
Apparent test prevalence	0.46 (0.39, 0.55)	0.33 (0.24, 0.44)
True sample prevalence	0.32 (0.25, 0.40)	0.25 (0.17, 0.35)
Accuracy (95% CI)	0.55 (0.47, 0.63)	0.66 (0.55, 0.75)
Sensitivity	0.52 (0.37, 0.66)	0.48 (0.28, 0.69)
Specificity	0.56 (0.46, 0.66)	0.72 (0.60, 0.81)
PPV	0.18 (0.14, 0.24)	0.24 (0.16, 0.36)
NPV	0.86 (0.81, 0.90)	0.88 (0.83,0.92)

**Table 4:** Inter-reviewer reliability

		Reviewer 2			
	OSA	No	Yes	Missing	Total
**Reviewer 1**	No	43	1	40	84
	Yes	23	32	18	73
	Missing	–	–	5	5
	Total	66	33	63	162

Note: Although computed agreement is high (76%) among the *n* = 99 that had their assessments on same individuals done, indicating better than average inter-rater reliability, after accounting for chance agreement using Cohen’s kappa, the kappa value is 0.53 and can be regarded as indicative of inter-rater moderate (kappa values of 0.41–0.60) agreement for the classification that was used. No = Chambe et al. [8] score < 2; Yes = Chambe et al. [8] score ≥ 2; Missing = subjects not scored

## Discussion

We determined the sensitivity of a FES-based screening tool for OSA in perioperative patients and hypothesized that it would have a minimum sensitivity of 70%. After analysis, sensitivity was 52% (95% CI: 37–66%) using a FES diagnostic cut-off of grade 2 or higher [8]. STOP-BANG scores were not calculated for patients with OSA diagnosed by sleep study. Thus, we did not directly compare the sensitivity and specificity of the STOP-BANG and FES-based screening tools within our sample population. However, the STOP-BANG questionnaire has been validated in meta-analysis with a pooled sensitivity of 84% when predicting any severity of OSA (95% CI: 81–87%) (14). The NPV of the STOP-BANG for OSA [14] was 56% (49–62%). We found an NPV of FES for OSA of 86% (81–90%) and 88% (83–92%) between both reviewers 1 and 2, respectively, indicating that using eyelid retraction to screen for FES has perioperative clinical utility. PPV was low at 18% (14–24%) and 24% (16–36%), likely because non-OSA and OSA groups were pre-diagnosed regarding OSA.

The specificity (56%, 95% CI: 46%, 66%) overlapped with the 95% CI of the meta-analysis that reported a STOP-BANG pooled specificity for any-OSA of 43% (95% CI: 38%, 49%) [14]. The FES-based screening tool proposed here had significantly lower sensitivity (95% CI 46.65%, 62.72%) and similar specificity when compared to STOP-BANG literature.

FES prevalence was higher in patients with (52%) than those without known OSA and a STOP-BANG < 3 (44%). This is consistent with prevalence found in a meta-analysis that showed a pooled OR of 4.12 for FES in OSA [11].

The gold standard diagnostic tool for OSA is the overnight polysomnogram. It is of little utility in the setting of urgent or emergent surgery. The STOP-BANG questionnaire was developed to provide a reliable screening tool for identifying patients at risk for OSA [13] and used perioperatively to prepare for possible airway and ventilation complications during procedures. The STOP-BANG questionnaire is recommended for detecting all OSA levels over the STOP questionnaire, Berlin Questionnaire, and Epworth Sleepiness Scale [15]. Despite high sensitivity, the STOP-BANG relies on reliable patient history and examination, which may not be available in emergent situations. Furthermore, the questionnaire requires the patient to relay subjective information about snoring and fatigue as well as an observer able to identify apnea periods. Similar issues arise with the popular Berlin questionnaire [15].

An objective screening tool which does not rely on patient history would be of utility in urgent and emergent settings. In addition, it would prompt more in-depth evaluation for OSA in the general population. Some studies have analyzed biomarkers as a screening tool for undiagnosed OSA; however, no single biomarker has been found to have sufficient diagnostic strength [16, 17]. In one study, hemoglobin A1c (HbA1c) plus C-reactive protein (CRP) plus erythropoietin (EPO) was found to be superior to the Epworth Sleepiness Scale and STOP-BANG questionnaire in screening for OSA [18]. However, delay for laboratory testing is not practical in the acute setting.

Another option would be the “OSA Score” developed by Friedman et al. that included BMI, modified Mallampati grade, and tonsil size [19]. In one study, the OSA Score, using a score cut-off of ≥ 6 to predict AHI ≥ 5 (mild or higher OSA), had a sensitivity of 86.3% and a specificity of 46.8% [6]. Despite high sensitivity of the OSA score, the Mallampati score cannot be assessed in unconscious or uncooperative patients, thus rendering this tool ineffective in the trauma setting. This is an issue because, in the trauma setting, suspicion of OSA may change therapy. A patient suspected of OSA may be sent to the ICU postoperatively or require close monitoring, resulting in increased resource utilization and cost.

Per meta-analysis, incidence of FES in OSA increases with OSA severity, with increased OR values of 2.56, 4.62, and 7.64 for mild, moderate, and severe OSA, respectively [11]. In the present study, participants with diagnosed OSA were also on CPAP therapy. The extent to which CPAP therapy affects the clinical course of eyelid laxity remains uncertain. Kadyan et al. showed no difference in eyelid laxity between CPAP and non-CPAP users despite better tear film break-up times and less ocular irritation in CPAP users [20]. McNab reported a case of a patient with complete reversal of FES after 4 years of treatment for OSA, despite maintaining a BMI of 39 for the duration of therapy [21]. Acar et al. further showed there was a significant decrease in FES diagnosed after PAP therapy in 51 patients (74.5% before PAP and 56.9% after PAP, *p* < 0.01) [22].

In conclusion, the findings of this study were encouraging for further research to address the connection of lid laxity and FES to OSA, and therefore to increased risk of perioperative complications. No singular OSA screening tool exists for patients unable to participate in such an assessment, and screening for lid laxity and the degree of FES may serve as that tool. Some limitations of this study include a small sample size as a pilot study, lack of randomization, and absence of an anesthesiologist as a scoring judge to evaluate how well anesthesiologists can identify grades of lid laxity compared to ophthalmologists in a clinical setting. In addition, participants in the non-OSA group, although at low risk for OSA, could not be guaranteed to not eventually have a positive diagnosis of OSA. This was not a diagnostic study, and patients did not have follow-up sleep studies with nocturnal oximetry or polysomnography to eliminate this bias between the groups. Further studies are needed, including those with recruitment of an experimental group of subjects with suspected but undiagnosed OSA, a more standardized approach for eyelid distraction, longitudinal follow-up to document any perioperative complications or subsequent diagnosis of OSA, as well as a cohort of perioperative clinicians in order to evaluate their skill at identification of lid laxity.

## Financial Disclosures

Departmental funds were provided for this study by the Department of Anesthesiology at the University of Nebraska Medical Center.
